# Reduced Neuronal Transcription of *Escargot*, the *Drosophila* Gene Encoding a Snail-Type Transcription Factor, Promotes Longevity

**DOI:** 10.3389/fgene.2018.00151

**Published:** 2018-04-30

**Authors:** Alexander V. Symonenko, Natalia V. Roshina, Anna V. Krementsova, Elena G. Pasyukova

**Affiliations:** ^1^Laboratory of Genome Variation, Institute of Molecular Genetics, Russian Academy of Sciences, Moscow, Russia; ^2^Laboratory of Genetic Basis of Biodiversity, N. I. Vavilov Institute of General Genetics, Russian Academy of Sciences, Moscow, Russia; ^3^Laboratory of Kinetics and Mechanisms of Enzymatic and Catalytic Reactions, N. M. Emanuel Institute of Biochemical Physics, Russian Academy of Sciences, Moscow, Russia

**Keywords:** *Drosophila melanogaster*, life span, longevity, aging, the nervous system, transcription factor, transcription

## Abstract

In recent years, several genes involved in complex neuron specification networks have been shown to control life span. However, information on these genes is scattered, and studies to discover new neuronal genes and gene cascades contributing to life span control are needed, especially because of the recognized role of the nervous system in governing homeostasis, aging, and longevity. Previously, we demonstrated that several genes that encode RNA polymerase II transcription factors and that are involved in the development of the nervous system affect life span in *Drosophila melanogaster*. Among other genes, *escargot* (*esg*) was demonstrated to be causally associated with an increase in the life span of male flies. Here, we present new data on the role of *esg* in life span control. We show that *esg* affects the life spans of both mated and unmated males and females to varying degrees. By analyzing the survival and locomotion of the *esg* mutants, we demonstrate that *esg* is involved in the control of aging. We show that increased longevity is caused by decreased *esg* transcription. In particular, we demonstrate that *esg* knockdown in the nervous system increased life span, directly establishing the involvement of the neuronal *esg* function in life span control. Our data invite attention to the mechanisms regulating the *esg* transcription rate, which is changed by insertions of DNA fragments of different sizes downstream of the structural part of the gene, indicating the direction of further research. Our data agree with the previously made suggestion that alterations in gene expression during development might affect adult lifespan, due to epigenetic patterns inherited in cell lineages or predetermined during the development of the structural and functional properties of the nervous system.

## Introduction

The nervous system is a key player in maintaining homeostasis and the structural and functional integrity of living beings and, hence, in controlling aging and longevity (Alcedo et al., 2013). The nervous system is a network of specialized neuronal cells, and their identity is established during development and maintained throughout adulthood due to the expression of genes coding for neurotransmitters and neuropeptides, ion channels, receptor and motor proteins, recognition and adhesion molecules, and other neuronal genes (Hobert, 2011). Given the role of the nervous system in life span control, a reasonable question would be whether genes defining the cellular specificity of neurons are also involved, in some way, in the regulation of longevity. Some of these genes have been shown to affect life span (Pasyukova et al., 2015). For example, *paralytic*, which encodes the major voltage-gated sodium channel and *maleless*, which encodes the ATP-dependent, double-stranded RNA helicase required for adenosine-to-inosine RNA editing and the proper expression of *paralytic*, are involved in both the regulation of synaptic activity (Reenan et al., 2000; Zhong and Wu, 2004) and in longevity control (Reenan and Rogina, 2008). A loss-of-function mutation in the *Odorant receptor co-receptor* gene that encodes a co-receptor protein participating in the sensory perception of smell (Mukunda et al., 2014) and the *Gustatory receptor 63a* that encodes the gustatory receptor that participates in the detection of carbon dioxide (Kwon et al., 2007) extend longevity (Libert et al., 2007; Poon et al., 2010). However, information on the role of neuronal genes in life span control remains scarce.

We have already demonstrated that several genes that encode RNA polymerase II transcription factors and that are involved in neural development affect life span in *Drosophila melanogaster* (Pasyukova et al., 2004; Rybina and Pasyukova, 2010; Roshina et al., 2014; Rybina et al., 2017). Among other genes, *escargot* (*esg*) was identified as a candidate gene affecting life span in a screen of more than 1,500 insertion mutations and the insertion located downstream of *esg* was further confirmed to be causally associated with life span control (Magwire et al., 2010). *esg* (http://flybase.org/reports/FBgn0001981.html) is localized on the second chromosome, and its single exon encodes an RNA polymerase II transcription factor that possesses five Zn^2+^-finger DNA-binding domains and a P-DLS-K domain; it can both repress and activate transcription by binding to the consensus DNA sequence 5′-A/GCAGGTG-3′ or to the dCtBP co-repressor (Ashraf et al., 1999; Ashraf and Ip, 2001; Cai et al., 2001). *esg* belongs to the Snail family of genes that are involved in the development of the nervous system in arthropods and chordates (Manzanares et al., 2001). In *Drosophila melanogaster*, Esg and other Snail proteins act to control asymmetric neuroblast division during embryogenesis; however, Esg functions are not exclusively neuronal, and it also participates in the maintenance of intestinal and male germ cells, regulates tracheal morphogenesis and development of the genital disk, and determines wing cell fate (http://flybase.org/reports/FBgn0001981.html).

Here, we present new data on the role of *esg* in life span control. Analysis of the *esg*^*BG01042*^ mutation allowed us to show that *esg* is involved in the regulation of life span, to varying degrees, in unmated and mated males and females. The *esg*^*BG01042*^ mutation also increased locomotion, specifically during old age, indicating that the mutation slowed down aging. The increase in longevity was caused by decreased *esg* transcription associated with structural changes in the DNA sequences downstream of the gene. This result was corroborated by the fact that the decrease in the *esg* transcription rate due to the gene-specific RNAi knockdown in the nervous system increased life span, directly establishing the involvement of the neuronal function of *esg* in life span control.

## Materials and methods

### Fly strains and crosses

The *w[1118]; P{GT1}esg*^*BG01042*^ (esgP) line is a homozygous *Drosophila melanogaster* line with a 8.5 kb *P{GT1}* insertion located 602 bp downstream of the *esg* gene in a *w*^*1118*^*(F)* (Control M) line background (http://flypush.imgen.bcm.tmc.edu/pscreen/transposons.html; Bellen et al., 2004; see also Magwire et al., 2010). Both lines were obtained from Trudy Mackay (North Carolina State University, USA).

The *w*^*1118*^; *P{GD1437}v9793* (Kdw1) line (estimated off-target effect is 4%; http://www.genomernai.org/v17/reagentdetails/9793) was used to provide *esg* RNAi knockdown; knockdown; the *w*^*1118*^ (Control K1) line without a transgene providing RNAi was used as a control line for the *esg* RNAi knockdown, as suggested by the manufacturer. Both lines were produced by and obtained from Vienna *Drosophila* Resource Center (Dietzl et al., 2007; http://stockcenter.vdrc.at/control/main).

Several lines were obtained from the Bloomington *Drosophila* Stock Center (USA) (http://flystocks.bio.indiana.edu/).

The *y*^1^ *v*^1^*; P{TRiP.JF03134}attP2* (Kdw2) and *y*^1^ *v*^1^*; P{TRiP.HMS00025}attP2* (Kdw3) lines were used to provide *esg* RNAi knockdown (estimated off-target effects are 0%; http://www.genomernai.org/v17/reagentdetails/DRSC03530; http://www.genomernai.org/v17/reagentdetails/DRSC37545); the *y*^1^ *v*^1^*; P{y*^+*t*7.7^ = *CaryP}attP2* (Control K2) line without a transgene providing RNAi was used as a control line for *esg* RNAi knockdown, as suggested by the manufacturer (http://flystocks.bio.indiana.edu/Browse/TRiPtb.htm).

The *P{w*^+*mW*.*hs*^ = *GawB}elav*^*C155*^ *w*^*1118*^*; P{w*^+*mC*^ = *UAS-Dcr-2.D}2* line was used to induce the expression of transgenic constructs in the nervous system. This driver line proved to be effective in our previous work, according to the real time RT-qPCR data (Rybina et al., 2017; Symonenko, unpublished results).

To induce expression of transgenic constructs, females of the driver line were crossed to males of Control K1, Control K2, Kdw1, Kdw2, and Kdw3 lines. Hybrid progeny used for life span measurements were further denoted as: Control K1, Control K2, Kdw1, Kdw2, and Kdw3. Life spans of hybrid Kdw1 individuals were compared to life spans of hybrid Control K1 individuals; life spans of hybrid Kdw2 and Kdw3 individuals were compared to life spans of hybrid Control K2 individuals. Dicer was present in all, both Control and Kdw, individuals.

Flies were kept at 25°C on a medium of semolina, sugar, raisins, yeast and agar with nipagin, propionic acid and streptomycin. For all experiments, the flies were collected from cultures with moderately controlled density: in each vial, 10–15 fertilized females of approximately the same age (5–20 days old) were allowed to lay eggs for 4 days.

### Tests for *Wolbachia*

Prior to the experiments, all the lines were checked for the presence of *Wolbachia*, a *Drosophila* symbiont known to affect life history traits (McGraw and O'Neill, 2004), via quantitative PCR (MiniOpticon real-time PCR detection system, Bio-Rad) with primers for the 16S rRNA gene, 5′-CATACCTATTCGAAGGGATAG-3′, and 5′-AGCTTCGAGTGAAACCAATTC-3′ (Werren and Windsor, 2000). Negative results were obtained for all lines except Control K1. This line was treated with tetracycline (0.25 mg/mL, Holden et al., 1993) for three generations followed by at least three generations of recovery, before it was used in experiments.

### PCR and sequencing

DNA was extracted from batches of 20 flies of each genotype using a standard phenol-chloroform procedure (Sambrook et al., 1989). DNA was used in PCR reactions with esg1 5′ AGTCAATTCCTATTTCCGGC 3′ and esg2 5′ CACCCGAACGATACCTTACC 3′ primers (expected product size of 508 bp). PCR products were sequenced on an ABI PRIZM 310 Genetic Analyzer (Applied Biosystems) using the esg1 and esg2 primers and a Big Dye Terminator V. 3.1. Kit (Applied Biosystems), according to the manufacturer's protocol.

### Life span assays

Life span was measured as described by Roshina et al. (2014). To measure the life span of unmated flies, 5 virgin flies of the same genotype and sex, all collected on the same day from cultures with moderate density, were placed in replicate vials. To measure the life span of mated flies, 3 virgin males and 3 virgin females of the same genotype were placed together in replicate vials. Flies were transferred to vials with fresh food containing approximately 5 mL of standard medium without live yeast on the surface weekly (virgin flies) or two times a week (mated flies). The number of dead flies was recorded daily. Experiments comparing fly life spans were conducted simultaneously. Sample sizes were 49–150 flies per sex per genotype. The experiments that showed noteworthy results were repeated two to five times. Five experiments with unmated flies were done for five consecutive years. Life spans of mated males and females were measured twice with an interval of approximately 6 months. Life spans of flies with *esg* knockdown were measured twice for two consecutive years. The life span for each fly was estimated as the number of days alive from the day of eclosion to the day of death. Mean and median life span and survival curves were primarily used to characterize life span.

### Locomotion assays

Locomotion was measured as described by Roshina et al. (2014). Flies were collected and maintained by the same procedures as for the life span assays but without recording the deaths. Locomotion was measured, at the same time each day, in unmated males and females and in mated males at age 1, 10, 20, 30, 40, or 50 days. Experiments comparing locomotion were conducted simultaneously. Sample sizes were 33–100 flies (11–20 vials) per genotype per age. One day before the measurements, five virgin flies of the same sex, age and genotype or three mated flies of the same sex, age and genotype were placed in the replicate vials. After every measurement, the mated males were returned to the vials with the females. To measure locomotor activity, the vials were placed horizontally in a *Drosophila* Population Monitor (TriKinetics). Fly movement along the walls or in the middle of the vial crossed the infrared beam rings along the length of the vial. Beam interruptions were detected and totals were reported every 5 min to the host computer. Two measurements for 5 min were made for each vial. Locomotion was characterized as the mean number of beam interruptions per vial.

### Real-time RT-qPCR

Total RNA for real-time reverse transcription quantitative PCR (RT-qPCR) was extracted from batches of 20 whole bodies of 1-, 10-, and 20-day-old virgin males, each batch collected from cohorts of different progeny, and from 50 embryos aged 14–20 h. For extractions, TRIzol reagent (Invitrogen) and DNase I (Sigma-Aldrich) were used according to the manufacturers' instructions.

First-strand cDNA was synthesized using SuperScript II Reverse Transcriptase (Invitrogen) with oligo(dT)_15_ primers according to the manufacturer's instructions. Amounts of cDNA were determined by RT-qPCR using SYBR Green I in a MiniOpticon real-time PCR detection system (Bio-Rad).

*Gdh* and *Adh* housekeeping genes, characterized by relatively low expression comparable to *esg* expression, were used as reference genes to normalize for differences in total cDNA between the samples. The forward and reverse primer sequences used were: Esg-rt1 5′-CGAGTTCTACAGGACCATCAATCAGC-3′ and Esg-rt2 5′-CGCCGATTGGTCTATGGATGAT-3′; *Gdh*: Gdh1 5′-TATGCCACCGAGCACCAGATTCC-3′ and Gdh2 5′-GGATGCCCTTCACCTTCTGCTTCTT-3′; *Adh*: Adhd3: 5′-CGGCATCTAAGAAGTGATACTCCCAAAA-3′ and Adhr3: 5′-TGAGTGTGCATCGAATCAGCCTTATT-3′.

CFX Manager 3.1 software (Bio-Rad, 2012) was used to evaluate the relative gene expression. Inter-run calibrations were used for each panel of experiments since the experiments were conducted for several years and two different models of Bio-Rad qPCR detection systems were used. Two to three independent RNA extractions (biological repeats) per genotype per age (developmental stage) were made. From three to six technical repeats were made for each RNA extraction, including, in some cases, independent c-DNA samples. The averaged results of technical repeats were used for further analysis.

### Statistical analyses

To compare control and mutant genotypes, Student's *t*-test and the nonparametric, distribution-free Kruskal-Wallis test were used for the analyses of locomotion and the amount of *esg* transcript. These two tests gave consistent results, so only the results of the Kruskal-Wallis test are reported here. Standard descriptive statistical analysis of life span (Wilmoth and Horiuchi, 1999; Carey, 2003) was performed to determine the mean life span and its accompanying variances, standard deviations and standard errors; the median, minimum and maximum life spans; and the life spans of the lower and upper quartiles, 10 and 90 percentiles (Supplementary Table). Survival curves were estimated using the Kaplan–Meier procedure. The nonparametric, distribution-free Mann-Whitney test and Kolmogorov-Smirnov test were used to evaluate the statistical significance of the difference between the survival curves. The Tukey test was used for multiple comparisons when appropriate.

## Results

### *esg^*BG01042*^* increased male and female life spans and locomotion

**Figure 1:**
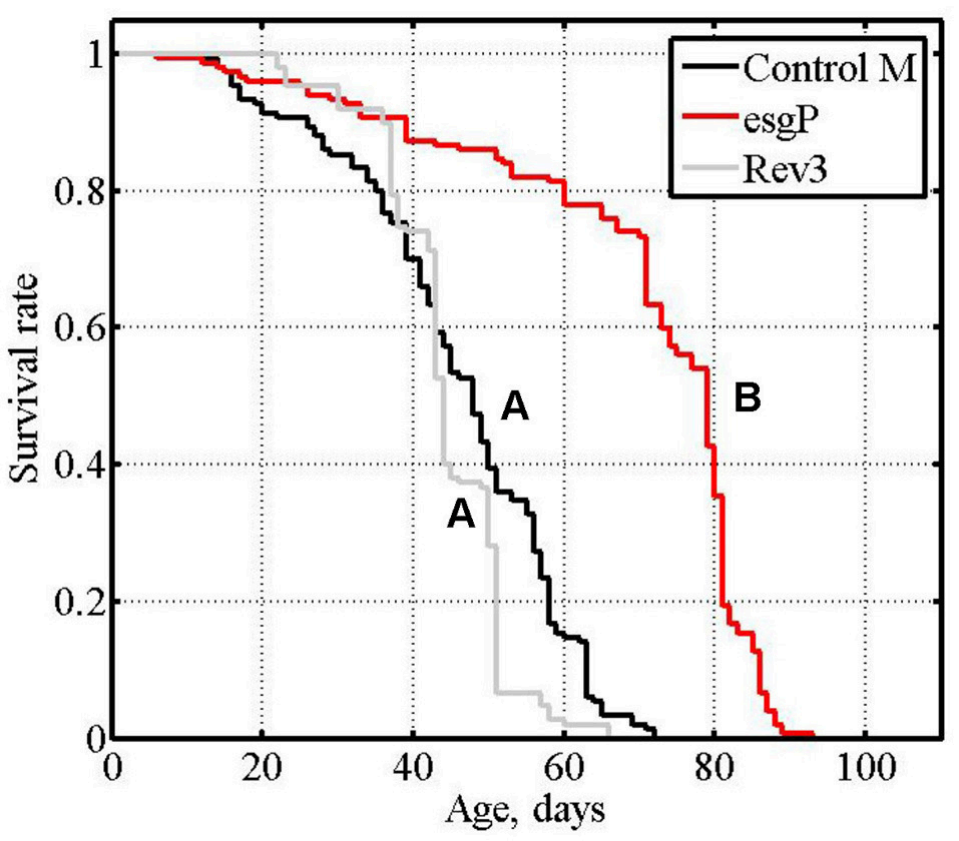
Initial analysis of mutant and revertant lines: survival of unmated males, cumulative data of experiments #1 and #2. Letters A and B indicate the results of Tukey tests for significant differences between control, mutant and revertant lines. Lines with the same letter are not significantly different from each other.

A *Drosophila melanogaster* line with the *esg* mutation, *w[1118]; P{GT1}esg*^*BG01042*^ (esgP) and a control line (Control M) with the genotype *w*^*1118*^ were used in this study to describe the impact of the gene in the control of life span and aging. The life spans of esgP and Control M flies were assessed several times. In the two screening experiments performed in the Trudy Mackay laboratory and described in Magwire et al. (2010), the life spans of esgP males were significantly longer than those of the controls, whereas the effect in females was not significant. This result was confirmed in the first experiment and the increase in length of the esgP male life span was further confirmed in the second experiment, performed in our laboratory (Magwire et al., 2010). The results of the two later experiments were presented in Magwire et al. (2010) only as a diagram showing the mean life spans. Here, we present these data in more detail for comparison with the other results; the distributive statistics are given in Table 1 and Supplementary Table (exp. #1 and #2) and the survival curves are shown in Figure 1.

**Table 1:** Distributive statistics of life span.

**Exp. no**	**Line**	**N**	**Mean**	**Standard error**	**Median**	**Percentile 90**	***P*****-values for comparisons with the corresponding control lines**		**Tukey test**
							**Mann-Witney test**	**Kolmogorov-Smirnov test**	
***esg*** **MUTATION, UNMATED MALES**									
1	Control	50	50	1.9	54	61			A
	esgP	50	61	2.1	63	77	<**0.0001**	<**0.001**	B
	rev3	49	57	2.8	52	77	0.7029	<**0.005**	A
	rev5	49	65	1.8	66	78	<**0.0001**	<**0.001**	B
2	Control	150	46	1.2	48	63			A
	esgP	150	70	1.6	79	86	<**0.0001**	<**0.001**	B
	rev3	150	44	0.7	44	51	0.0840	<**0.005**	A
3	Control M	100	36	1.4	38	53			
	esgP	100	57	1.9	66	72	**0.0001**	<**0.001**	
4	Control M	150	28	1.1	26	48			A
	esgP	150	55	1.3	58	71	**0.0001**	<**0.001**	B
	Control M × esgP	50	45	2.6	52	63	**0.0006**	<**0.005**	C
5	Control M	195	45	1.2	48	61			A
	esgP	110	66	1.5	69	83	**0.0001**	<**0.001**	B
	Rev3	150	49	1.4	48	76	0.1856	< 0.1	A
	Rev5	205	56	1.2	59	77	**0.0001**	<**0.001**	C
***esg*** **MUTATION, UNMATED FEMALES**									
1	Control M	50	62	2.1	67	77			
	esgP	50	65	1.8	67	83	0.5533	>0.10	
3	Control M	100	36	1.7	38	53			
	esgP	100	48	1.6	55	65	**0.0001**	<**0.001**	
4	Control M	150	41	1.2	45	59			A
	esgP	150	51	1.5	56	72	**0.0001**	<**0.001**	B
	Control M × esgP	50	49	2.2	54	63	**0.0003**	<**0.001**	C
5	Control M	114	45	1.5	47	66			A
	esgP	124	58	1.6	61	77	**0.0001**	<**0.001**	B
	Rev3	114	50	1.9	51	76	0.0243	< 0.1	A
	Rev5	115	56	1.3	54	72	**0.0001**	<**0.001**	B
***esg*** **MUTATION, MATED MALES**									
6	Control M	60	30	1.9	30	50			
	esgP	60	46	2.2	50	66	**0.0001**	<**0.001**	
7	Control M	60	33	1.5	36	45			
	esgP	60	44	2.3	46	67	**0.0001**	<**0.001**	
***esg*** **MUTATION, MATED FEMALES**									
6	Control M	60	37	1.6	38	50			
	esgP	60	37	1.9	37	57	0.9581	>0.1	
7	Control M	60	34	1.4	37	45			
	esgP	60	34	1.9	34	50	0.8011	>0.1	
***esg*** **RNA-i KNOCKDOWN IN THE NERVOUS SYSTEM, UNMATED MALES**									
8	Control K2	100	51	1.6	52	70			
	Kdw2	100	47	0.9	46	59	**0.0112**	<**0.005**	
	Kdw3	100	58	1.8	58	79	**0.0021**	<**0.025**	
9	Control 2	100	61	1.5	64	74			
	Kdw3	100	65	1.7	67	85	**0.0351**	<**0.05**	
***esg*** **RNA-i KNOCKDOWN IN THE NERVOUS SYSTEM, UNMATED FEMALES**									
8	Control K2	100	76	2.0	82	97			
	Kdw2	100	71	2.1	77	92	**0.0111**	< 0.1	
	Kdw3	100	84	1.6	90	98	**0.0004**	<**0.001**	
9	Control K2	100	88	1.4	92	101			
	Kdw3	100	93	1.3	95	105	**0.0049**	<**0.025**	

*N is a number of flies in the experiment; Median indicates a number of days when 50% of flies are dead; Percentile 90 indicates a number of days when 90% of flies are dead. Standard errors were calculated from the variance among vials. Significant P-values are in bold case. When multiple lines were compared, an underscored line indicates significant P-values that did not survive Bonferroni correction. The mean life spans and survival curves of Control M and esgP flies from experiments 1 and 2 have already been published (Magwire et al., 2010). Control M, the w^1118^(F) line where the P{GT1} insertion in the 3′ regulatory region of esg was obtained obtained (Magwire et al., 2010). esgP, the line with an esg^BG01042^ mutation in the w^1118^(F) background. Rev3, the line with the precise P{GT1} excision. Rev5, the line with the imprecise P{GT1} excision. Control K2, the y^1^ v^1^; P{y^+t7.7^ = CaryP}attP2 line proposed as a control line for Kdw2 and Kdw3 by the manufacturer (http://flystocks.bio.indiana.edu/Browse/TRiPtb.htm). Kdw2, the y^1^ v^1^; P{TRiP.JF03134}attP2 line used to provide esg RNAi knockdown. Kdw3, the y^1^ v^1^; line used to provide esg RNAi knockdown*.

**Figure 2:**
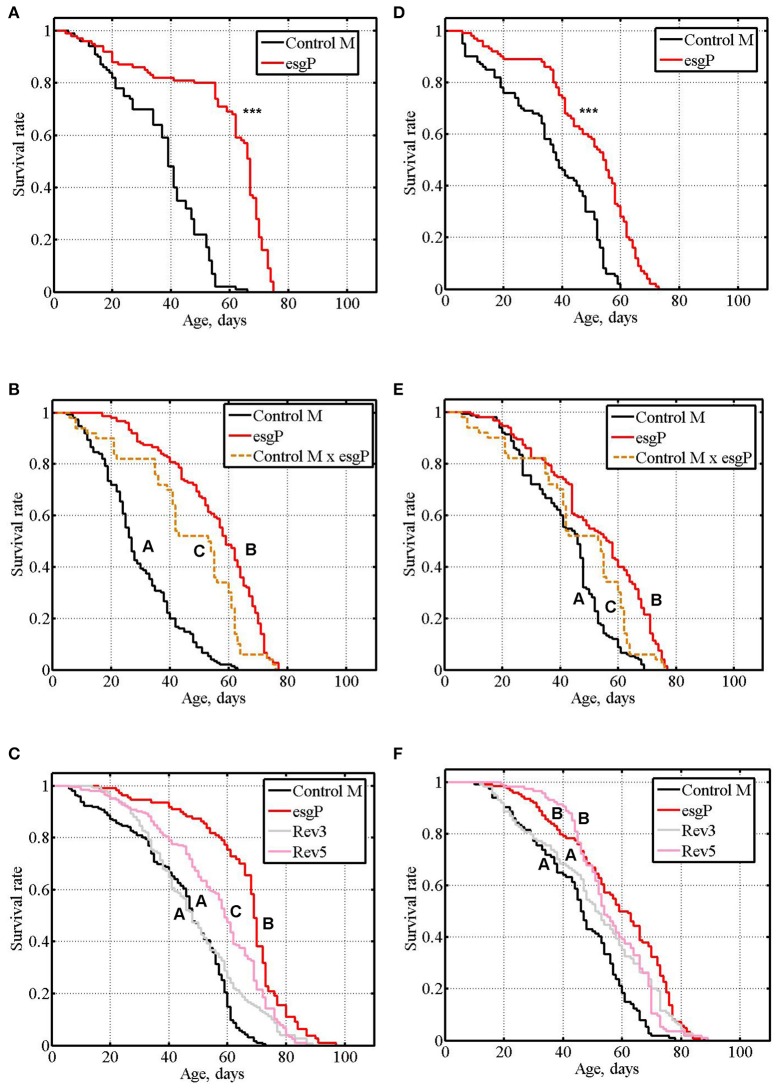
Survival of unmated mutant and revertant flies. **(A–C)**: experiments #3, #4, #5 with males. **(D–F)**: experiments #3, #4, #5 with females. Asterisks denote significant differences with the Control M line, as determined by the Mann-Whitney test (^***^P < 0.001). Letters A, B, and C indicate the results of Tukey tests for significant differences between different genotypes. Genotypes with the same letter are not significantly different from each other.

Sex-specific and sex-antagonistic effects on life span are not rare (Tricoire et al., 2009; Ruiz et al., 2011; Roshina et al., 2014; Schriner et al., 2014; Shaposhnikov et al., 2015); for example, of 59 mutations increasing life span, 70.7% affected males and females differently (Magwire et al., 2010). In the initial screens and in our first experiment (Magwire et al., 2010), the effects of *esg*^*BG01042*^ on the life span of female flies were not statistically significant leading to the conclusion that *esg*^*BG01042*^ affects life span in a male-specific manner. However, the sample sizes in these experiments were small, and weak effects could have gone unnoticed. To better understand whether the effects of *esg*^*BG01042*^ on life span are strictly male-specific, we re-measured life span in esgP and Control M males and females (Table 1 and Supplementary Table, exp. #3; Figures 2A,D). A significant increase in life span was detected in mutant males compared with control males as well as in mutant females compared with control females. Nevertheless, the positive effect of the mutation was considerably smaller in females (33% of the control mean life span) than in males (58%). This result was confirmed in two other experiments (Table 1 and Supplementary Table, exp. #4 and #5; Figures 2B,C,E,F). Overall, the positive effect of the mutation varied from 22 to 96% in males and from 4 to 33% in females, the average effects being 55 and 17%, respectively. Comparison of survival curves in all experiments indicated that the mutation slowed aging in both males and females (Figure 2). The maximum life span of mutant males was 40% higher and the maximum life span of mutant females was 17% higher than the controls (Table 1).

The life span of heterozygous esgP/Control M males and females was significantly higher compared to homozygous Control M flies and significantly lower compared to esgP flies (Table 1 and Supplementary Table, exp. #4; Figures 2B,E), which indicated a co-dominance of the *esg*^*BG01042*^ mutation.

**Figure 3:**
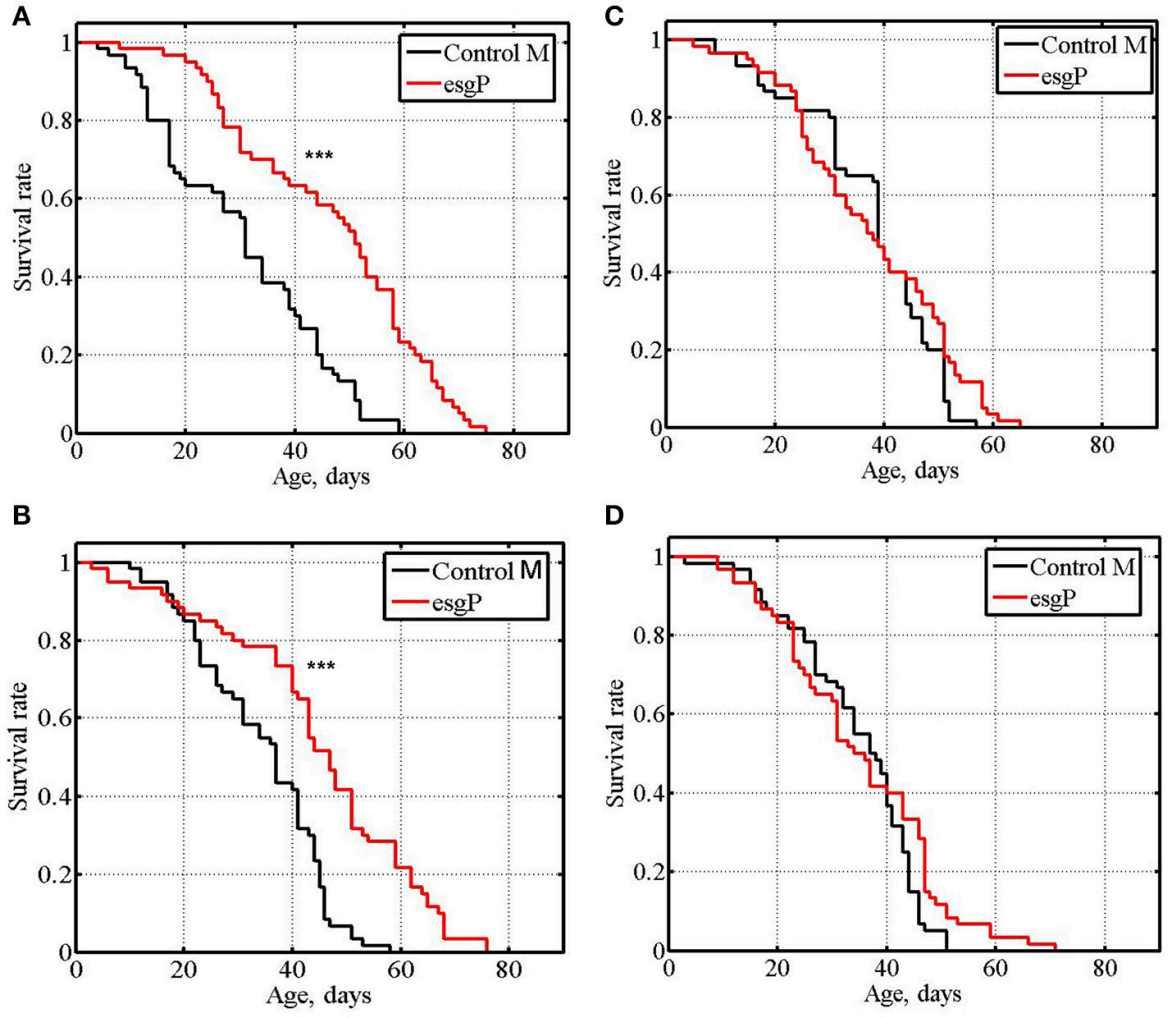
Survival of mated mutant flies. **(A,B)**: experiments #6, #7 with males. **(C,D)**: experiments #6, #7 with females. Asterisks denote significant differences with the Control M line as determined by the Mann-Whitney test (^***^P < 0.001).

To further assess the effects of *esg*^*BG01042*^ on life span, we compared the life spans of mated esgP and Control M flies (Table 1 and Supplementary Table, exp #6, exp. #7). While, technically, experiments with unmated flies are easier, mated flies represent a better model of the naturally occurring way of life. A significantly increased life span was detected in mated mutant males compared with control males (Figure 3A). To verify this result, the experiment was repeated and the life span of mutant males was again significantly higher than that of control males (Figure 3B). The life span of mutant mated females was not different from the life span of control females (Figure 3C). To verify this result, the experiment was repeated and identical results were obtained (Figure 3D). In mated males, the average positive effect of the mutation was somewhat lower (43%) than that in unmated males. A comparison of the survival curves in experiments with mated males indicated that the mutation slowed aging (Figures 3A,B). In mutant males, the maximum life span was 40% higher, on average, than that of the controls (Table 1).

**Figure 4:**
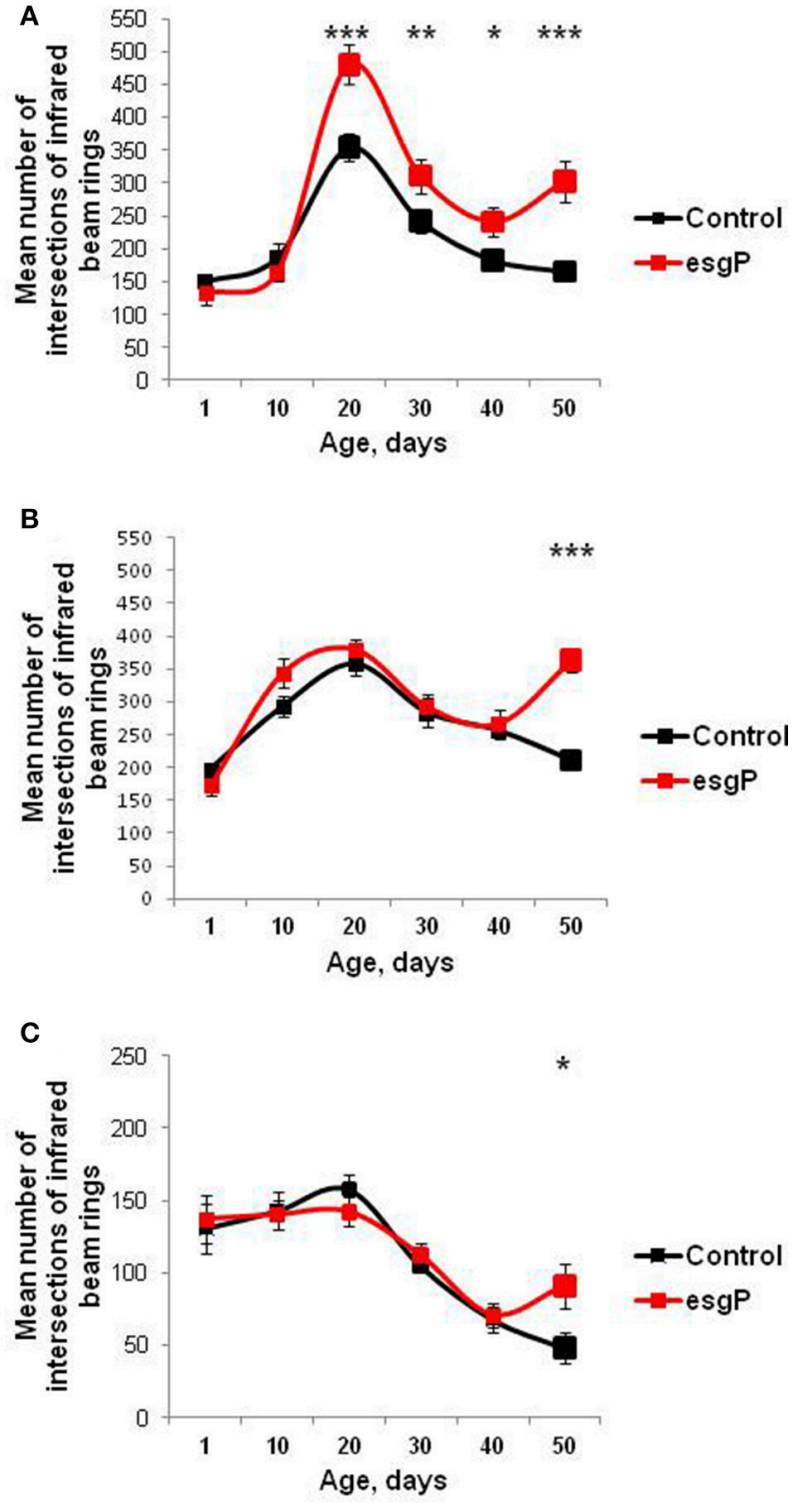
Age-dependent locomotion of mutant flies. **(A)** Locomotion of unmated males **(B)** Locomotion of unmated females. **(C)** Locomotion of mated males. Asterisks denote significant differences compared with the Control M line, as determined by the Kruskall-Wallis test (^*^P < 0.05; ^**^P < 0.01; ^***^P < 0.001).

General locomotor activity decreases with normal aging in animals and is often considered a marker of age and health (Ridgel and Ritzmann, 2005). In unmated males and females and in mated males, survival curves indicated that the mutation slowed down aging. We assessed the effect of *esg*^*BG01042*^ on locomotor activity in unmated males and females and mated males to double check if the rate of aging is actually affected in these cases. After the 20th day of life, an age-dependent decline in locomotor activity was observed in both the mutant and control flies, regardless of their mating status, in good agreement with our prediction (Figure 4). However, in mutant flies with an increased life span, this age-dependent decline was followed by an elevation in locomotion recorded in the 50-day-old individuals compared with the controls (Figure 4). Locomotion was significantly increased in all 50-day-old mutant flies with an increased life span, compared with the controls. These results indicate that, in old mutant flies, aging was effectively slowed down or reversed. In addition, in mutant males, in which the mutation affected life span most strongly, locomotion increased starting at 20 days (Figure 4). Overall, the effects of locomotion paralleled those of life span, and a higher level of mobility in older mutant flies confirmed our hypothesis, which was based on an initial comparison of survival curves, that *esg*^*BG01042*^ would slow aging.

### *esg^*BG01042*^* decreases *esg* transcript amounts

**Figure 5:**
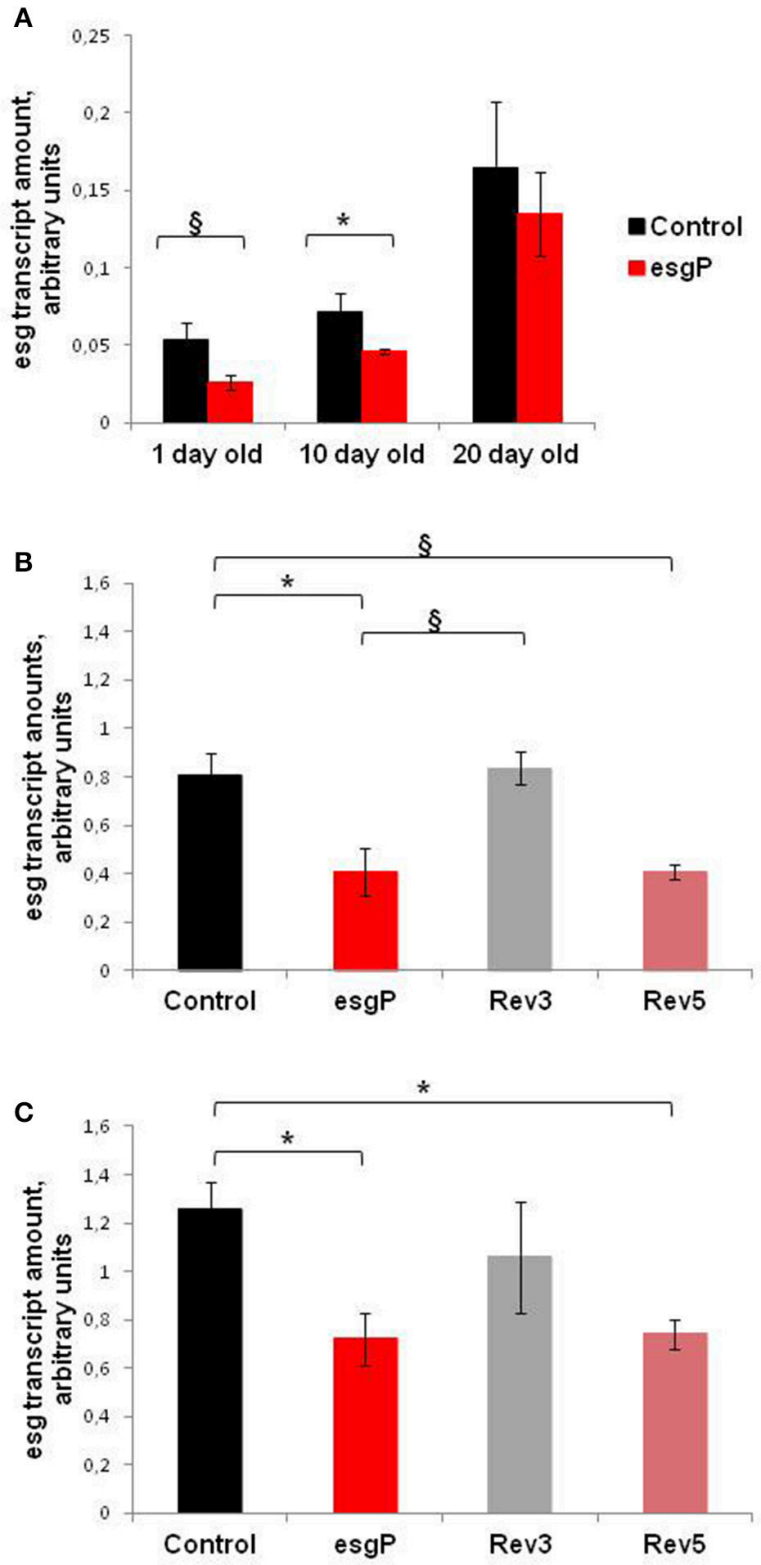
*esg* transcript amounts in unmated mutant and revertant flies and embryos. Transcript amounts in **(A)** unmated males of different ages; **(B)** 1 day-old unmated males; **(C)** embryos. Two biological repeats were done in the first experiment with 1- and 20- day-old males **(A)**. Three biological repeats were done in the first experiment with 10-day-old males **(A)**, in the second experiment with 1-day-old males **(B)**, and in the experiment with embryos **(C)**. The paragraph sign denotes marginally significant differences (^§^*P* < 0.10) and the asterisk denotes significant differences (^*^P < 0.05) as determined by the Kruskall-Wallis test.

To understand the molecular basis of differences in life span caused by the mutation, we assessed the effect of *esg*^*BG01042*^ on *esg* transcript amounts (Figure 5). Only one protein coding *esg* transcript and one polypeptide have been reported (http://flybase.org/reports/FBgn0001981.html). Transcript amounts of *esg* in adult flies are very low and are slightly higher in males than in females due to transcription in testes (http://flybase.org/reports/FBgn0001981.html). The effects of *esg*^*BG01042*^ on life span were the strongest in unmated males. For these reasons, we concentrated our main efforts on measuring transcription rates in unmated males. In our initial experiment conducted simultaneously with experiment #3 measuring life span, we compared total *esg* transcript amounts in 1-, 10- and 20-day-old unmated males with and without the mutation. A significant decrease in *esg* transcript amounts was found in 10-day-old mutant males compared with controls (Figure 5A). In 1-day-old mutant males, the increase in expression was marginally significant and in 20-day-old males the increase was not statistically significant. Our overall interpretation of these results was that *esg*^*BG01042*^ reduced *esg* transcript amounts at all ages but, in some cases, quantities of mRNA were too small to reach the level of statistical significance. The statistically significant differences were confirmed when comparing *esg* transcript amounts in 1-day-old males in the experiment that was conducted a year later, at the same time with experiment #4 measuring life span (Figure 5B).

We made an attempt to measure transcription rates in 1-day-old control and mutant unmated females. Several technical replicates made with the same biological samples demonstrated that the amount of *esg* RNA is slightly smaller in mutants (0.9 ± 0.02 vs. 1 ± 0.02 relative arbitrary units). Given small absolute amounts of *esg* mRNA in females and a small difference observed in this single experiment, we did not pursue this line of study any more. Satisfactory enough, the result of the experiment with females was in line with our conclusion that the *esg* transcription rate is lower in mutants than in controls.

Expression of *esg* is predominant in embryos (http://flybase.org/reports/FBgn0001981.html). The expression of *esg* mRNA is persistent throughout embryogenesis, with the highest amounts observed from 2 up to 20 h of development. Simultaneously with experiment #5, we evaluated *esg* transcript amounts in 14–20-h-old mutant and control embryos. This moderately large interval allowed us to obtain the overall characteristics of control and mutant embryos and to offset the possible uneven contribution of embryos at different stages of development. The *esg* transcript amounts were significantly lower in mutant embryos than in control embryos (Figure 5C).

### Precise and imprecise reversions of *esg^*BG01042*^* have different effects on life span and *esg* expression

**Figure 6:**
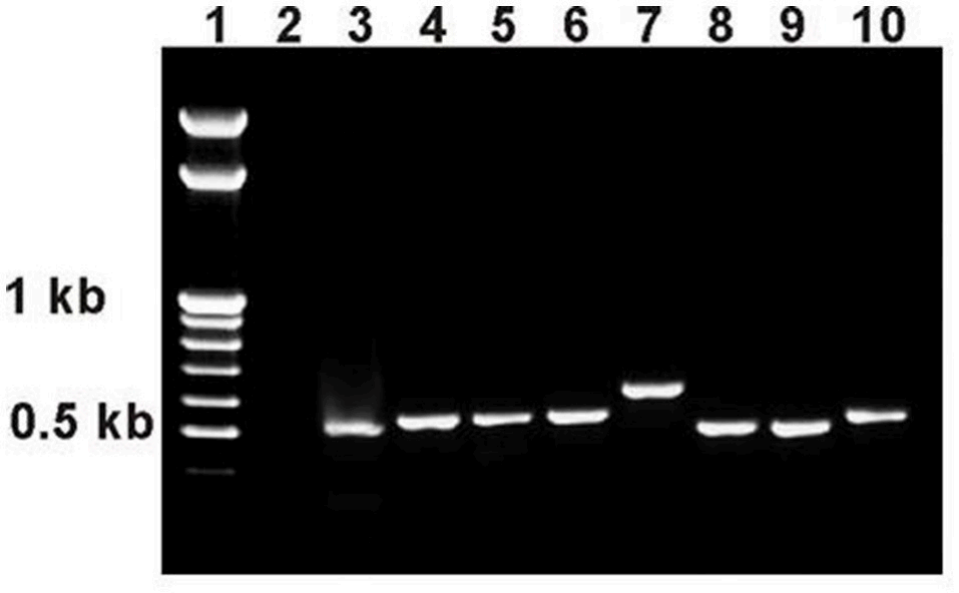
PCR analysis of *esg* structure in revertant lines. PCR primers esg1 and esg2 were used with an expected product size of 508 bp. 1: marker (GeneRuler™ 100 bp Mass DNA Ladder, Fermentas); 2: esgP (negative control, an expected PCR product of approximately 9 kb is not produced under the PCR conditions used); 3: Control M; 4: Rev1.1; 5: Rev1.2; 6: Rev2.1; 7: Rev2.2; 8: Rev3; 9: Rev4; 10: Rev5.

Standard substitution crosses with balancers and delta 2–3 source of P element transposase (Robertson et al., 1988) were used to obtain lines with reversions of the *esg*^*BG01042*^ mutation, while maintaining the co-isogenic background. These crosses were described in more detail in Magwire et al. (2010), the detailed description of revertant lines is given here. Seven lines with reversions of the marker *w*^+^ phenotype were obtained from five males with active transposase. For each line, PCR with primers flanking the site of the initial *P{GT1}* insertion were used to assess the nature of the reversions. PCR fragment sizes were identical in the control line and in two lines with reversions, indicating precise excisions of the *P{GT1}* construct. In five other lines with reversions, the PCR fragment sizes were slightly larger, indicating that imprecise excisions occurred (Figure 6). This was further confirmed by sequencing the PCR fragments: in one line (Rev3), the sequence was identical to the standard gene sequence (http://flybase.org/reports/FBgn0001981.html), in the other line with a precise excision (Rev4), three nucleotide substitutions were detected; in lines Rev1.1, Rev1.2, Rev2.1, Rev2.2, and Rev5, insertions of 30–162 bp in length were detected between the duplicated insertion sites. Two revertant lines, Rev3 with the complete restoration of the original genome structure and Rev5 with the 32 bp insertion 602 bp downstream of *esg*, were kept in stock and used in this study.

The life span of Rev3 males was measured in experiments #1 and #2 to demonstrate that the reversion of the mutation was accompanied by the reversion of the mutant phenotype. The life span of Rev3 males was the same as that of Control M males, and the results of Tukey tests for significant differences between the control, mutant and revertant lines proved the causal relationship between the mutation and life span changes (Magwire et al., 2010). Experiment #5 confirmed the effect of the precise reversion on the male life span and attested that the same effect was observed in females (Table 1, Figures 2C,F).

The life span of Rev5 males was measured in experiments #1 and #5 and the life span of Rev5 females was measured in experiment #5 to compare the effects of precise and imprecise reversions. In experiment #1, the life span of Rev5 males was not different from that of esgP males (*P* = 0.1564 for Mann-Whitney test). The Tukey test allowed us to divide the lines into two groups: Control M and Rev3 vs. esgP and Rev5 (Table 1). In experiment #5, the life span of Rev5 males was different from both esgP males (*P* < 0.0001 for the Mann-Witney test) and control M males (Table 1). The esgP and Rev5 lines divided into different groups, while the Control M and Rev3 lines remained in the same group (Table 1, Figure 2C). The life span of Rev5 females was not different from the life span of esgP females (*P* = 0.1075 for the Mann-Witney test), and the Tukey test allowed us to divide the lines into two groups: Control M and Rev3 vs. esgP and Rev5 (Table 1, Figure 2F). The results indicated that a small 32 bp insertion located 602 bp downstream of *esg* had virtually the same effect on male and female life spans as a large 8500 bp *P{GT1}* insertion.

This conclusion raised the important question of whether the differences and similarities in life spans in control, mutant and revertant lines were paralleled by the differences and similarities in esg transcription levels. We evaluated *esg* transcript amounts in mutant, control, and revertant 1-day-old males and in 14- to 20-h-old embryos (Figures 5B,C). In neither case was the difference significant between the Control M and Rev3 lines or between the esgP and Rev5 lines. The difference between the Control M and Rev5 was marginally significant in males and significant in embryos; the difference between esgP and Rev3 was marginally significant in males. Visually, Control M and Rev3 formed one group and esgP and Rev5 formed a second group, however, the Tukey test did not confirm this. Despite some statistical failures, these results indicate that the precise reversion of the mutation was accompanied by the reversion of the transcription level, while the imprecise reversion did not affect the transcription level characteristic for the mutation. This gives us serious reasons to say that a decrease in *esg* transcription underlies the decrease in the life span of mutants. Additionally, these results confirmed that a small 32 bp insertion and a large 8.5 kb *P{GT1}* insertion located 602 bp downstream of *esg* affects *esg* expression in the same way.

### *esg* knockdown in the nervous system increases male and female life span

Analysis of the *esg* mutation and its precise reversion demonstrated that the decrease in *esg* transcription was causally associated with the increase in life span. Transcript levels were measured, either in the whole bodies of adult flies or in whole embryos, and what changes in which tissues were responsible for the observed effects remains unknown. Given that *esg* mRNA is not abundant and, hence, precise evaluation of its quantity in individual tissues of mutant and control flies is rather difficult technically, we used the binary GAL4-UAS system to induce RNAi knockdown of *esg* and in this way to decrease its expression in a tissue-specific manner. To get a broader understanding of the knockdown effects, we used three independent lines with RNA interference in the hope that at least one of them would provide a decrease in *esg* transcription similar to the effect of the mutation. We were primarily interested in understanding the role of neuronal genes and the nervous system in life span control. Based on this, we decided to evaluate the effects of the *esg* knockdown in the nervous system on male and female life spans. Life span of unmated males and females was selected as a tester trait in these experiments because, technically, experiments with unmated flies are easier and the effects generally tend to be more obvious. Also, both unmated males and females demonstrated response to changes in the *esg* function, whereas among mated flies, only males were affected.

*w*^*1118*^*; P{GD1437}v9793, y*^1^ *v*^1^*; P{TRiP.JF03134}attP2, y*^1^ *v*^1^*;* and *P{TRiP.HMS00025}attP2* lines with transgenes encoding *esg* hairpins, and the corresponding control lines suggested by the manufacturers were used to evaluate the effects of the knockdown. A well-known line, well-known line, *P{w*^+*mW*.*hs*^ = *GawB}elav*^*C155*^ *w*^*1118*^*; P{w*^+*mC*^ = *UAS-Dcr-2.D}2*, was used to induce the expression of transgenic constructs in all the neurons. The presence of an additional, inducible copy of *Dicer* in its genome provided better interference. Hybrid progeny used for life span measurements were denoted as Kdw1, Kdw2, Kdw3, Control K1, and Control K2.

**Figure 7:**
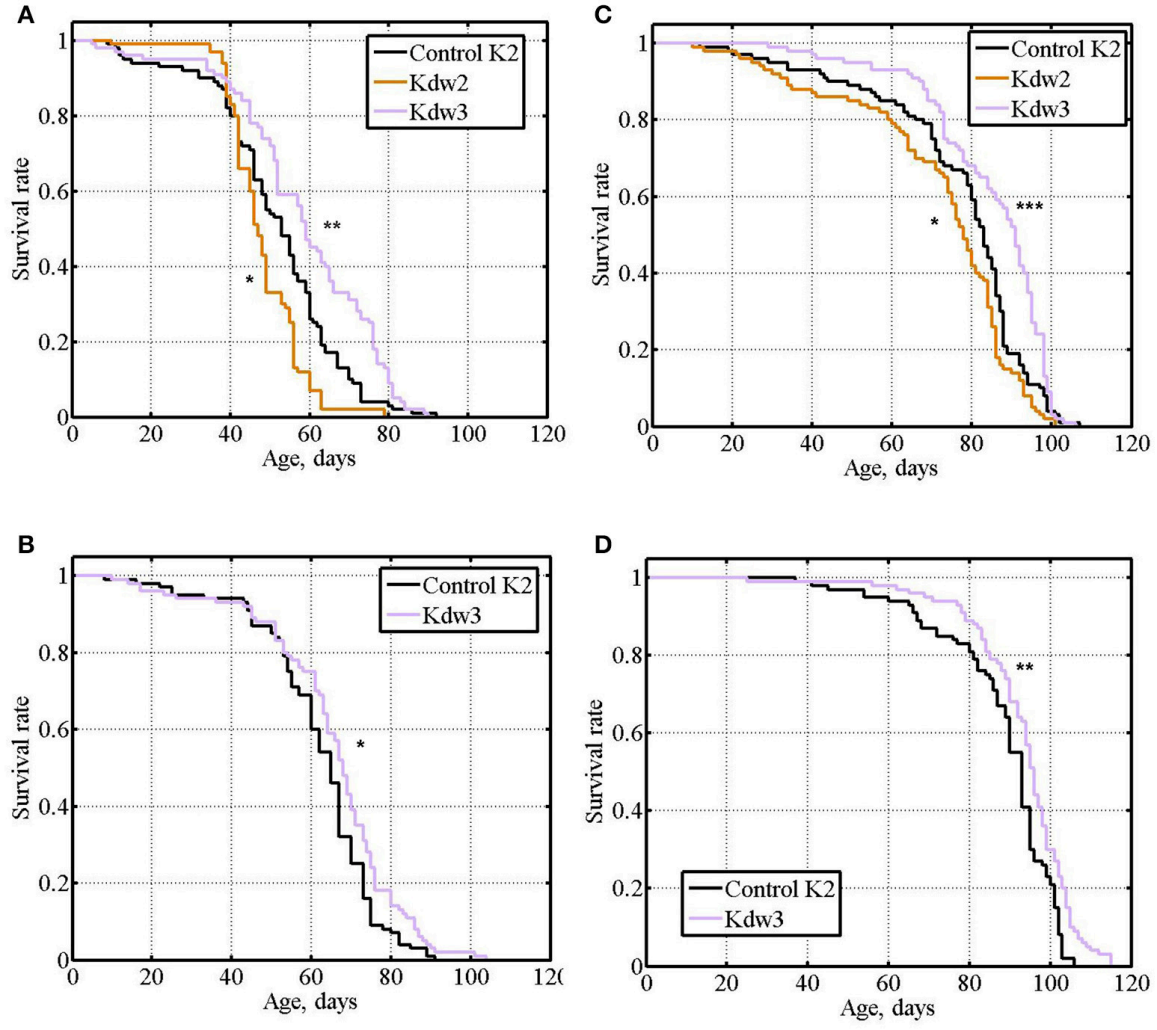
Survival of unmated flies with *esg* knockdown in the nervous system. **(A,B)**: experiments #8, #9 with males. **(C,D)**: experiments #8, #9 with females. Asterisks denote significant differences with the Control K2 line as determined by the Mann-Witney test (^*^P < 0.05; ^**^P < 0.01; ^***^P < 0.001).

In experiment #8, different results were obtained with the three lines providing *esg* knockdown. In the first case (Kdw1), *esg* knockdown in the nervous system caused lethality. In the second case (Kdw2), in both males and females, life span was significantly lower compared with the controls (Table 1 and Supplementary Table, Figures 7A,C). In the third case (Kdw3), in both males and females, life span was significantly higher compared with the controls (Table 1 and Supplementary Table, Figures 7A,C). In the two later cases differences were rather small. The positive effect was reproduced in experiment #9 (Table 1 and Supplementary Table, Figures 7B,D).

Our results demonstrated that reduced *esg* expression in the nervous system may cause both deleterious and beneficial effects. It would be logical to suggest that the strength and the direction of the effect depend on the effectiveness of the knockdown provided by different lines. Kdw1 might provide the strongest knockdown and the lowest rate of *esg* transcription incompatible with survival, Kdw2 might provide an intermediate level of knockdown, and Kdw3 might provide the slightest level of knockdown, resulting in a beneficial fine-tuning of the level of *esg* transcription. However, according to the manufacturer's description, Kdw 3 is supposed to provide a stronger knockdown than Kdw2 (http://flystocks.bio.indiana.edu/Browse/RNAi/RNAi_D_M.php). A rather specific suggestion that an intermediate level of *esg* transcription in the nervous system is the most beneficial for longevity might account for the results we obtained. A similar hypothesis explained our data on the effects of *Lim3* expression on life span (Rybina and Pasyukova, 2010; Rybina et al., 2017). Unfortunately, the level of *esg* expression both in the central nervous system (heads) and peripheral nervous system (carcasses) of adult flies is extremely low (http://flybase.org/reports/FBgn0001981.html), which makes the direct evaluation of the knockdown effects in these tissues with a precision necessary for statistically reliable comparisons rather tricky.

Overall, the data obtained in the experiments with *esg* knockdown corroborated data obtained in the experiments with the *esg* mutation: a certain level of decline in *esg* expression had beneficial effects on survival and aging.

## Discussion

### *esg* affected *Drosophila* longevity

In this study we demonstrated that *esg*, a gene encoding a Snail-type transcription factor, increased life span and slowed down aging. The *esg* mutation most strongly affected unmated males, and, to a lesser extent mated males and unmated females. It was not detected at all in mated females. We also demonstrated that the precise reversion of the *esg* mutation was associated with the restoration of the control phenotype, both in males and in females, and thus confirmed the causal relationships between the mutation and the life span changes reported earlier (Magwire et al., 2010). However, effects of this single mutation, *esg*^*BG01042*^, on life span are not formally sufficient to assert that *esg* is involved in life span control. Indeed, it was shown that the insertion located far enough downstream from the structural part of the gene is causally associated with life span, but whether this insertion affects the function of this particular gene remained unknown. Microarray analysis failed to reveal changes in *esg* transcription rate in *esg*^*BG01042*^ mutants, which could be explained by insufficient power of the method (Magwire et al., 2010). We demonstrated that *esg*^*BG01042*^ affected gene transcription rate and proved that the decrease in *esg* expression is causally related to the life span increase. This is a crucial result which for the first time demonstrated that *esg* is involved in life span control.

Though there is no doubt that the *P{GT1}* insertion affected *esg* expression, there remained a possibility that it also affected expression of another gene located downstream of *esg* and that it was this change that caused the observed increase in life span. However, there are no genes close to *esg*, the nearest downstream neighbor is CG15258, located at a distance of 27,055 bp. Information about this gene is scarce; it is predominantly expressed in the embryonic central nervous system, embryonic/larval midgut and adult testes (http://flybase.org/reports/FBgn0032563.html). Given the distance between the *P{GT1}* insertion and CG15258, remote effects of *P{GT1}* on CG15258 expression seem improbable, though we cannot fully exclude them.

Determining if other *esg* mutations have effects on fly longevity could be suggested in order to confirm the involvement of the gene in lifespan control. Several insertion mutations affecting *esg* are publicly available (http://flybase.org/reports/FBgn0001981.html). However, the answer to this question is already known. In the initial screen of mutations affecting life span (Magwire et al., 2010), seven other insertion mutations in *esg* affected life span, though in the opposite direction. All seven mutations were in different locations from the mutation associated with increased life span. Mutations decreasing life span are generally assumed to be deleterious and affect various life history traits including fitness, while mutations increasing life span are likely to have specific effects on the trait and, as such, are more interesting for the study of the genetic control of life span. Still, the fact that eight independent mutations in *esg* affect life span reinforces the conclusion that this gene is involved in the control of life span. Experiments with gene-specific knockdowns provided final, independent proof of the involvement of *esg* in life span control.

Of note, the only *esg* mutation associated with an increased life span was in a slightly different genetic background compared with the mutations associated with decreased life span, which indicate unknown epistatic interactions (Magwire et al., 2010). This evidence agrees with our data showing that the mutations in *shuttle craft* had different effects on life span in different genetic backgrounds (Pasyukova et al., 2004; Roshina et al., 2014) and the data from other researchers. For example, naturally segregating genes interact epistatically with *Sod*, a well-known aging gene, to modify its ability to extend longevity (Spencer et al., 2003). The genetic basis of these epistatic interactions remains unknown, but, obviously, *esg* is expected to interact with other genes because it encodes a transcription factor.

Transcriptional cascades are key regulatory mechanisms (Skeath and Thor, 2003; Jothi et al., 2009). Transcription factors such as FOXO, HSF-1, HIF-1, NFkB, TFEB, and others participate in different signaling cascades and are crucial for the regulation of longevity and aging (for reviews see Alcedo et al., 2013; Bhatt and Ghosh, 2014; Lapierre et al., 2015). Previously, we demonstrated that two genes, *shuttle craft* and *Lim3*, which encode transcription factors involved in the development of the nervous system, affect *Drosophila* life span (Pasyukova et al., 2004; Roshina et al., 2014; Rybina et al., 2017). New data from this study adds one more gene to this list.

Our results demonstrated that a decrease in *esg* transcription in the nervous system prolonged life span, thus confirming that the neuronal function of *esg* is indeed relevant to life span control. Microarray analysis revealed approximately 100 *esg* primary targets whose transcription was induced or repressed by neuronal *esg* overexpression in *Drosophila* larvae (Hekmat-Scafe et al., 2005). Targets of *esg* encoded enzymes involved in the biosynthesis of neurotransmitters, neuropeptides, cationic transporters and other proteins. Among others, genes involved in the defense/immune response were both up- and down-regulated. Of the genes known to be involved in life span control, at least two genes associated with increased life span, *heat shock protein 26 (hsp26)* and *NAD-dependent methylenetetrahydrofolate dehydrogenase* (*Nmdmc*), increased transcription (Wang et al., 2004; Yu et al., 2015). It is unclear whether transcription of a similar pool of genes would be affected by a reduction in *esg* expression or whether the same effects would be observed at other developmental stages and in adults. Obviously, an opposite effect, if any, on *hsp26* and *Nmdmc* transcription would be expected upon *esg* knockdown, which means that changes in the expression of other *esg* targets should lead to increased life span in this case, or that *esg* effects in *hsp26* and *Nmdmc* in embryos and adults would be different compared to in larvae.

In our study, *esg* knockdown affected the nervous system at all stages of development. It remained undetermined at which stage a decrease in *esg* transcription was essential for an increase in life span. Normally, *esg* transcription is low in adult heads and carcasses, which comprise the central and peripheral nervous systems (http://flybase.org/reports/FBgn0001981.html), and its further reduction by knockdown probably reduces it almost to zero. To the best of our knowledge, nothing is known about the vital *esg* neuronal functions in adult flies, and though it does not mean that these functions do not exist, this fact emphasizes that reducing *esg* transcription to a virtually negligible level in the adult nervous system is not deleterious. The best known neuronal function of Esg and other Snail family proteins is to control asymmetric neuroblast division during embryonic development (Ashraf and Ip, 2001; Cai et al., 2001, 2003; Wodarz and Huttner, 2003) via two distinct highly conserved mechanisms: one functions throughout mitosis and is implemented via the control of *inscuteable* and *string*, the other acts during anaphase/telophase and is *inscuteable*-independent. There are data indicating that during embryogenesis *esg* has other functions related to the development of the nervous system (Hartl et al., 2011; Kim et al., 2015; Ramat et al., 2016). One might suggest that it is a decrease in *esg* transcription at the developmental stages, in particular, at the embryonic stage that matters for longevity. We previously suggested that the long-term, carry-over effects of alterations in embryonic gene expression might be epigenetically inherited in cell lineages or, alternatively, might affect transcriptional cascades that predetermine structural and functional properties of the adult nervous system (Roshina et al., 2014). Our preliminary data showed that insertion mutations in genes that are also involved in the control of asymmetric neuroblast division, such as *inscuteable, shaggy*, and *aPKC*, are also able to increase life span; however, these results only represent the first steps toward investigating the molecular mechanisms underlying *esg* effects on life span. Interestingly, ectopic neuronal expression of *esg* is a general seizure suppressor and *esg* must be ectopically expressed during nervous system development to produce adults that are less seizure prone (Hekmat-Scafe et al., 2005). Our suggestions agree well with these data.

The decrease in *esg* transcription in the nervous system caused by RNAi knockdown affected life span to a much lesser degree than the decrease in *esg* transcription caused by the *esg* mutation. Evidently, in mutant flies, other tissues were also involved in the implementation of the effects of *esg* on life span. As a transcription factor, Esg affects many aspects of fly physiology. Effects of the *esg*^*BG01042*^ mutation on gene transcription were consistent with highly pleiotropic functions of *esg* (Magwire et al., 2010). However, *esg* transcription is predominantly limited to just a few tissues and organs: the nervous system (embryos and larvae), gastrointestinal tract (all stages), and testis (adults) (http://www.flymine.org/flymine/report.do?id=1040284&trail=%7c1040284). These general characteristics, however, do not shed light on the exact genetic and metabolic pathways underlying the changes in life span. In recent years, significant attention has been drawn to the fact that *esg* is expressed in stem cells in several tissues, including intestinal stem cells (ISCs), cyst stem cells and male germline stem cells and is a primary component of testis stem cells (Loza-Coll et al., 2014; Voog et al., 2014; Loza-Coll and Jones, 2016). The gastrointestinal tract of multicellular animals was recently recognized as an organ critically important for the control of homeostasis, life span, and aging (Jasper, 2015). In the adult midgut, Esg is expressed in ISCs, and loss of Esg causes their rapid differentiation, whereas an increase in Esg expression locks ISCs into a stem cell state (Korzelius et al., 2014; Loza-Coll et al., 2014). Esg is thus a major repressor specifying whether ISCs remain undifferentiated or commit to differentiation. We hypothesize that a slight decrease in *esg* expression, such as was observed in *esg*^*BG01042*^ mutants, might shift the ISCs metabolism toward differentiation, thus providing rejuvenation of the gastrointestinal tract and slowing down aging. Given that the effects of *esg*^*BG01042*^ on longevity were more pronounced in males, one might also suggest that similar rejuvenation observed in testes upon decrease in *esg* expression was responsible for the sex-specificity of aging of *esg* mutants. We also hypothesize that the accelerated rejuvenation of the germline stem cells in testes induced by *esg* mutation might have a more pronounced effect on the rate of aging in unmated males because crossing and accompanying expenditure of germ cells partially neutralizes the effect of the mutation in mated males. We thus suggest that the difference in effects of the mutation on life spans of unmated and mated males is rather connected to differences in their physiological and behavioral patterns than to differences in expression levels of *esg*. Further experiments will shed light on the suggested differential mechanisms of life span control.

### The role of the 3′ regulatory region in modulating *esg* transcription

Molecular mechanisms underlying the relationship between gene expression and phenotype are central to understanding the basics of development, life and aging. In this study, we demonstrated that a decrease in *esg* transcript levels caused by the insertion of a rather large vector construct 602 bp downstream of *esg* and 1,240 bp downstream of the end of the single *esg* exon increased survival and slowed aging. The insertion was located far enough from the structural part of the gene but it remains unclear how it affected transcription or the properties of the transcript that set up its stability. To add to the complexity, our results demonstrated that the size of the insertion (32 bp compared to approximately 8,500 bp) had a negligible effect on *esg* transcription, which indicated that it was disruption of the regular genomic sequence rather than the properties of the inserted fragment that affected *esg* expression. According to our analysis using databases of *Drosophila* regulatory elements (http://www.fruitfly.org/seq_tools/promoter.html; http://www.ifti.org/cgi-bin/ifti/Tfsitescan.pl; http://alggen.lsi.upc.es/cgi-bin/promo_v3/promo/promo.cgi?dirDB=TF_8.3), the presence of a 32 bp insertion did not add any putative regulatory sites (promoters, transcriptional regulators binding sites) to the regular genome sequence downstream of *esg*.

It is not yet well understood how downstream sequences affect gene transcription (Pance, 2013). Enhancers are able to exert their effects, both upstream and downstream, over long distances (Schaffner, 2015). They function as integrated transcription factor binding platforms (Spitz and Furlong, 2012) and are characterized by special chromatin features (Calo and Wysocka, 2013). Polycomb/Trithorax Response Elements (PRE/TREs) also function over long distances and represent protein-binding platforms that are required to maintain gene transcription and to provide epigenetic inheritance of silent and active chromatin states in cell lineages (Schuettengruber et al., 2007). The latter property was especially interesting in the context of suggested carry-over effects of alterations in *esg* transcription from embryos to adults.

A possible explanation of the crucial effect of the relatively small insertions on *esg* transcription rates is that they might perturb the integrity of hypothetical protein-binding platforms. According to the FlyBase analysis (FlyBase, 1992)^1^ of modENCODE data (The modENCODE Consortium et al., 2010) the insertion site co-localizes with a possible Transcription Factor Binding Motifs (TFBS) hotspot. At least one of the possible TFBSs (http://flybase.org/reports/FBsf0000298779.html) refers to the Dorsal transcription factor, which may act as an RNA polymerase II distal enhancer binding factor for *esg* (Bhaskar and Courey, 2002).

A brief analysis (http://alggen.lsi.upc.es/cgi-bin/promo_v3/promo/promo.cgi?dirDB=TF_8.3) revealed several putative regulatory sites ±250 bp from the insertion site of both *P{GT1}* and the 32 bp DNA fragment remaining after the imprecise excision of *P{GT1}*. Five proteins involved in control of neurogenesis, synaptic structure and function and three proteins involved in the regulation of sex determination, sexual behavior and fertility might potentially bind DNA sequences located around the insertion. Among others, Doublesex (DSX) deserved our special attention, as it was shown that the regulatory region comprising DSX binding site was able to direct fat body expression of the reporter gene located 8,000 bp upstream (Garabedian et al., 1986), that is, demonstrated properties of enhancer. It is possible to suggest that functionally related sites might form enhancer-like platforms whose integrity was violated by insertions, which led to a decrease in *esg* transcription.

Ten sites with DNA motifs shown to be important for PRE/TRE function (Ringrose and Paro, 2007) were found in this region, indicating a possibility that Zeste, Pleiohomeotic, GAGA Factor/Pipsqueak and Dorsal Switch Protein 1 might bind DNA in this region. However, the density of the putative binding sites of proteins that are characteristic for PRE/TREs was too low to predict a PRE/TRE presence in the region (Ringrose and Paro, 2007). Of note, Zeste was shown to function as an enhancer for *snail* (Fuse et al., 1999).

We fully recognize that, even the presence of several binding sites of the same protein as well as the presence of several binding sites of functionally related proteins does not always appear to have functional consequences. Thorough bioinformatic and functional analyses of the region are needed to shed light on the regulatory properties of the DNA sequences downstream of *esg*. Some results on this point have been already reported. According to the REDfly database (http://redfly.ccr.buffalo.edu/; Gallo et al., 2011) several cis-regulating modules (CRMs) were found downstream of the site of interest, the best shot being the CRM esg_MLC2 localized 2642 bp downstream of *esg* (Halfon, 2014) and presumably enhancing the transcription rate of target genes in the midline glia, median neuroblast and its progeny (Long et al., 2014). If our insertion moves it away, we may expect the *esg* transcription rate in the developing nervous system of the embryo to be downregulated, in good accordance with our implied model. A violation of the *esg*_MLC2 regulation of *esg* transcription rate seems quite probable in the case of a large, 8,500 bp insertion; however, its exact position relative to *esg* must be highly sensitive in order to admit the same effect as a small 32 bp insertion.

Other possible mechanisms involved in the downstream control of *esg* transcription, including tissue-specific transcription enhancement or repression and nucleosome shift or other types of chromatin remodeling, could be the focus of future studies.

## Conclusions

To conclude, in our current study we planned to find out the following: (1) to corroborate the influence of the *esg* mutation on life span and describe its effects in more detail; (2) to find out whether the mutation affects *esg* expression and how gene expression should change in order to increase life span; (3) to understand whether the neuronal function of the gene is important for this. We demonstrated that the *esg* mutation affects gene transcription rate and proved that the decrease in *esg* expression is causally related to the life span increase. This crucial result for the first time demonstrated that *esg* is involved in life span control. We used the *esg* mutation to characterize effects of the gene on the life span of flies of different sexes and physiological statuses. We demonstrated that the *esg* mutation affects both male and female life span; that life span of females is affected less than lifespan of males; that life spans of unmated flies are affected more than life spans of mated flies; that aging is slowed down in mutant flies. We suggested that functional changes in tissues where *esg* is expressed (the nervous system, the gastrointestinal tract, testes) might be responsible for the effect on life span. As we are predominantly interested in unraveling the role of neuronal genes in life span control, we subjected the role of the *esg* function in the nervous system to a special study. For this purpose, effects of *esg* knockdown in the nervous system on life span were characterized. The results of these experiments allowed us to prove that the decrease in *esg* expression exclusively in the nervous system is sufficient to increases life span. By directly establishing the involvement of the neuronal function of *esg* in life span control, we have progressed toward achieving our main goal: to set a collection of genes encoding neuronal transcription factors involved in the life span control. This collection now includes *shuttle craft* (Roshina et al., 2014), *Lim3* (Rybina et al., 2017) and *esg* and will be further used for analysis of molecular mechanisms underlying aging.

## Author contributions

AS and NR have designed experiments; collected and interpreted data; critically revised the manuscript. AK have designed experiments; analyzed and interpreted data; critically revised the manuscript. EP have created the concept for the work and designed experiments; analyzed and interpreted data; drafted and critically revised the manuscript. All authors have approved the version to be published.

### Conflict of interest statement

The authors declare that the research was conducted in the absence of any commercial or financial relationships that could be construed as a potential conflict of interest.

## Footnotes

^1^http://flybase.org/reports/FBgn0001981
