# Supplementary material for: Reduced Neuronal Transcription of Escargot, the Drosophila Gene Encoding a Snail-Type Transcription Factor, Promotes Longevity
Source: Front Genet. 2018 Apr 30;9:151. doi: 10.3389/fgene.2018.00151 (PMC5936762; doi:10.3389/fgene.2018.00151)
Supplement: Supplementary file 1 [file Table_1.doc]

Supplementary Table. Distributive statistics of life span.

| Exp. No | Line | | N | Mean | Median | Minimum | Maximum | Lower Quartile | Upper Quartille | Percentile 10 | Percentile 90 | Variance | Standard Deviation | Standard Error | P values for comparisons with the corresponding Control lines | | | Tukey test |
| --- | --- | --- | --- | --- | --- | --- | --- | --- | --- | --- | --- | --- | --- | --- | --- | --- | --- | --- |
|  |  |  |  |  |  |  |  |  |  |  |  |  |  |  | Mann-Witney test | Kolmogorov-Smirnov test | |  |
| ***esg* mutation, unmated males** | | | | | | | | | | | | | | | | | | |
| 1 | Control | | 50 | 50 | 54 | 11 | 77 | 47 | 58 | 28 | 61 | 182.5 | 13.5 | 1.9 |  | |  | A |
|  | esgP | | 50 | 61 | 63 | 5 | 88 | 55 | 69 | 41 | 77 | 220.3 | 14.8 | 2.1 | **<0.0001** | | **<0.001** | B |
|  | rev3 | | 49 | 57 | 52 | 7 | 81 | 37 | 71 | 30 | 77 | 383.4 | 19.6 | 2.8 | 0.7029 | | **<0.005** | A |
|  | rev5 | | 49 | 65 | 66 | 25 | 89 | 57 | 75 | 50 | 78 | 158.8 | 12.6 | 1.8 | **<0.0001** | | **<0.001** | B |
| 2 | Control | | 150 | 46 | 48 | 9 | 72 | 39 | 57 | 26 | 63 | 201.0 | 14.2 | 1.2 |  | |  | A |
|  | esgP | | 150 | 70 | 79 | 6 | 93 | 67 | 81 | 39 | 86 | 364.5 | 19.1 | 1.6 | **<0.0001** | | **<0.001** | B |
|  | rev3 | | 150 | 44 | 44 | 22 | 66 | 38 | 51 | 36 | 51 | 69.5 | 8.3 | 0.7 | **<0.0001** | | **<0.001** | A |
| 3 | Control M | | 100 | 36 | 38 | 4 | 65 | 24 | 47 | 14 | 53 | 207.2 | 14.4 | 1.4 |  | |  |  |
|  | esgP | | 100 | 57 | 66 | 3 | 74 | 54 | 69 | 19 | 72 | 379.1 | 19.5 | 1.9 | **0.0001** | | **<0.001** |  |
| 4 | Control M | | 150 | 28 | 26 | 4 | 62 | 18 | 38 | 11 | 48 | 179.3 | 13.4 | 1.1 |  | |  | A |
|  | esgP | | 150 | 55 | 58 | 16 | 76 | 43 | 67 | 28 | 71 | 242.2 | 15.6 | 1.3 | **0.0001** | | **<0.001** | B |
|  | Control M x esgP | | 50 | 45 | 52 | 5 | 75 | 35 | 60 | 17 | 63 | 337.9 | 18.4 | 2.6 | **0.0006** | | **<0.005** | C |
| 5 | Control M | | 195 | 45 | 48 | 6 | 73 | 34 | 59 | 18 | 61 | 281.2 | 16.8 | 1.2 |  | |  | A |
|  | esgP | | 110 | 66 | 69 | 16 | 97 | 61 | 73 | 45 | 83 | 237.4 | 15.4 | 1.5 | **0.0001** | | **<0.001** | B |
|  | Rev3 | | 150 | 49 | 48 | 14 | 89 | 35 | 61 | 27 | 76 | 309.4 | 17.6 | 1.4 | 0.1856 | | <0.1 | A |
|  | Rev5 | | 205 | 56 | 59 | 6 | 87 | 46 | 69 | 31 | 77 | 319.2 | 17.9 | 1.2 | **0.0001** | | **<0.001** | C |
| ***esg* mutation, unmated females** | | | | | | | | | | | | | | | | | | |
| 1 | Control M | | 50 | 62 | 67 | 24 | 91 | 55 | 70 | 37 | 77 | 219.4 | 14.8 | 2.1 |  | |  |  |
|  | esgP | | 50 | 65 | 67 | 38 | 89 | 56 | 71 | 45 | 83 | 162.8 | 12.8 | 1.8 | 0.5533 | | >0.10 |  |
| 3 | Control M | | 100 | 36 | 38 | 5 | 59 | 23 | 51 | 8 | 53 | 272.9 | 16.5 | 1.7 |  | |  |  |
|  | esgP | | 100 | 48 | 55 | 5 | 72 | 39 | 61 | 19 | 65 | 266.2 | 16.3 | 1.6 | **0.0001** | | **<0.001** |  |
| 4 | Control M | | 150 | 41 | 45 | 5 | 68 | 29 | 51 | 22 | 59 | 201.9 | 14.2 | 1.2 |  | |  | A |
|  | esgP | | 150 | 51 | 56 | 8 | 76 | 38 | 67 | 23 | 72 | 326.1 | 18.1 | 1.5 | **0.0001** | | **<0.001** | B |
|  | Control M x esgP | | 50 | 49 | 54 | 6 | 75 | 45 | 59 | 23 | 63 | 241.6 | 15.5 | 2.2 | **0.0003** | | **<0.001** | C |
| 5 | Control M | | 114 | 45 | 47 | 10 | 78 | 33 | 58 | 22 | 66 | 266.0 | 16.3 | 1.5 |  | |  | A |
|  | esgP | | 124 | 58 | 61 | 12 | 88 | 46 | 73 | 32 | 77 | 322.0 | 17.9 | 1.6 | **0.0001** | | **<0.001** | B |
|  | Rev3 | | 114 | 50 | 51 | 12 | 87 | 36 | 69 | 20 | 76 | 411.1 | 20.3 | 1.9 | 0.0243 | | <0.1 | A |
|  | Rev5 | | 115 | 56 | 54 | 19 | 89 | 46 | 69 | 41 | 72 | 184.7 | 13.6 | 1.3 | **0.0001** | | **<0.001** | B |
| ***esg* mutation, mated males** | | | | | | | | | | | | | | | | | | |
| 6 | Control M | 60 | | 30 | 30 | 3 | 58 | 16 | 43 | 11 | 50 | 223.7 | 15.0 | 1.9 |  | |  |  |
|  | esgP | 60 | | 46 | 50 | 7 | 74 | 29 | 58 | 24 | 66 | 287.3 | 16.9 | 2.2 | **0.0001** | | **<0.001** |  |
| 7 | Control M | 60 | | 33 | 36 | 9 | 57 | 22 | 43 | 17 | 45 | 136.0 | 11.7 | 1.5 |  | |  |  |
|  | esgP | 60 | | 44 | 46 | 2 | 75 | 36 | 58 | 17 | 67 | 316.0 | 17.8 | 2.3 | **0.0001** | | **<0.001** |  |
| ***esg* mutation, mated females** | | | | | | | | | | | | | | | | | | |
| 6 | Control M | 60 | | 37 | 38 | 8 | 56 | 30 | 46 | 16 | 50 | 150.4 | 12.3 | 1.6 |  | |  |  |
|  | esgP | 60 | | 37 | 37 | 4 | 64 | 24 | 50 | 19 | 57 | 207.7 | 14.4 | 1.9 | 0.9581 | | >0.1 |  |
| 7 | Control M | 60 | | 34 | 37 | 2 | 50 | 26 | 42 | 16 | 45 | 125.0 | 11.2 | 1.4 |  | |  |  |
|  | esgP | 60 | | 34 | 34 | 8 | 70 | 22 | 46 | 15 | 50 | 206.0 | 14.4 | 1.9 | 0.8011 | | >0.1 |  |
| ***esg* RNA-i knockdown in the nervous system, unmated males** | | | | | | | | | | | | | | | | | | |
| 8 | Control K2 | 100 | | 51 | 52 | 8 | 91 | 41 | 61 | 33 | 70 | 258.4 | 16.1 | 1.6 |  | |  |  |
|  | Kdw2 | 100 | | 47 | 46 | 9 | 78 | 41 | 55 | 38 | 59 | 87.0 | 9.3 | 0.9 | **0.0112** | | **<0.005** |  |
|  | Kdw3 | 100 | | 58 | 58 | 4 | 89 | 47 | 74 | 38 | 79 | 323.8 | 18.0 | 1.8 | **0.0021** | | **<0.025** |  |
| 9 | Control K2 | 100 | | 61 | 64 | 7 | 90 | 53 | 70 | 44 | 74 | 217.1 | 14.7 | 1.5 |  | |  |  |
|  | Kdw3 | 100 | | 65 | 67 | 9 | 103 | 59 | 74 | 44 | 85 | 300.0 | 17.3 | 1.7 | **0.0351** | | **<0.05** |  |
| ***esg* RNA-i knockdown in the nervous system, unmated females** | | | | | | | | | | | | | | | | | | |
| 8 | Control K2 | 100 | | 76 | 82 | 11 | 106 | 69 | 87 | 45 | 97 | 385.9 | 19.6 | 2.0 |  | |  |  |
|  | Kdw2 | 100 | | 71 | 77 | 9 | 100 | 63 | 85 | 33 | 92 | 444.7 | 21.1 | 2.1 | 0.0311 | | >0.1 |  |
|  | Kdw3 | 100 | | 84 | 90 | 28 | 105 | 72 | 95 | 66 | 98 | 249.1 | 15.8 | 1.6 | **0.0004** | | **<0.001** |  |
| 9 | Control K2 | 100 | | 88 | 92 | 36 | 105 | 84 | 98 | 66 | 101 | 205.2 | 14.3 | 1.4 |  | |  |  |
|  | Kdw3 | 100 | | 93 | 95 | 24 | 114 | 88 | 101 | 78 | 105 | 169.0 | 13.0 | 1.3 | **0.0049** | | **<0.025** |  |

N is a number of flies in the experiment; Median indicates a number of days when 50% of flies are dead; Lower Quartille indicates a number of days when 25% of flies are dead; Lower Quartille indicates a number of days when 25% of flies are alive; Percentile 10 indicates a number of days when 10% of flies are dead; Percentile 90 indicates a number of days when 90% of flies are dead. Standard errors were calculated from the variance among vials. Significant P-values are in bold case. When multiple lines were compared, an underscored line indicates significant P values that did not survive Bonferroni correction. The mean life spans and survival curves of Control M and esgP flies from experiments 1 and 2 have already been published (Magwire *et al*., 2010). Control M, the *w^1118^(F)* line where the *P{GT1}* insertion in the 3’ regulatory region of *esg* was obtained (Magwire et al., 2010). esgP, the line with an *esg^BG01042^* mutation in the *w^1118^(F)* background. Rev3, the line with the precise *P{GT1}* excision. Rev5, the line with the imprecise *P{GT1}* excision. Control K2, the *y^1^ v^1^; P{y^+t7.7^=CaryP}attP2* line proposed as a control line for Kdw2 and Kdw3 by the manufacturer (<http://flystocks.bio.indiana.edu/Browse/TRiPtb.htm>). Kdw2, the *y^1^ v^1^; P{TRiP.JF03134}attP2* line used to provide *esg* RNAi knockdown. Kdw3, the *y^1^ v^1^; P{TRiP.HMS00025}attP2* line used to provide *esg* RNAi knockdown.
